# Population Genetics of the European Roma—A Review

**DOI:** 10.3390/genes13112068

**Published:** 2022-11-08

**Authors:** Giacomo Francesco Ena, Julen Aizpurua-Iraola, Neus Font-Porterias, Francesc Calafell, David Comas

**Affiliations:** 1 Department of Medicine and Life Sciences (MELIS), Institute of Evolutionary Biology (CSIC-UPF), Universitat Pompeu Fabra, 08002 Barcelona, Spain; 2 Department of Biomedical Informatics, Anschutz Medical Campus, University of Colorado, Aurora, CO 80309, USA

**Keywords:** population genetics, genomics, Romani, genetic structure, bottleneck, founder event

## Abstract

The Roma are a group of populations with a common origin that share the Romani identity and cultural heritage. Their genetic history has been inferred through multiple studies based on uniparental and autosomal markers, and current genomic data have provided novel insights into their genetic background. This review was prompted by two factors: (i) new developments to estimate the genetic structure of the Roma at a fine-scale resolution have precisely identified the ancestral components and traced migrations that were previously documented only in historical sources, clarifying and solving debates on the origins and the diaspora of the Roma; (ii) while there has been an effort to review the health determinants of the Roma, the increasing literature on their population genetics has not been subjected to a dedicated review in the last two decades. We believe that a summary on the state of the art will benefit both the public and scholars that are approaching the subject.

## 1. Introduction

The Romani population constitute the largest transnational ethnic minority across the European continent, and while they are commonly known to be traditionally itinerant, most of them have been settled for centuries. Despite their long presence in numerous countries and the assimilation into part of the local culture to different extents, Romani groups share a common identity and cultural traditions [1,2]. For centuries, most of the information regarding Romani history originated from linguistics, written historical records from non-Romani populations and socio-anthropological comparisons, although recent insights have come from the characterization of their genetic background [3,4,5,6,7]. According to historical findings [2,8], this nomadic state of living, including cultural and social practices (for instance, endogamy) that are uncommon in the dominant European populations, has led to a cultural friction which very often resulted in social marginalization and persecution. Due to this, a significant number of the Roma might have decided not to register their ethnicity in official censuses [9]. In 2019, Romani people in Europe were estimated to number 10 million [10]; however, some Romani and international human rights organizations declare that they exceed 14 million, meaning that they constitute 1.5–1.8% of the total European population [10]. Most Romani populations are established in South-Eastern Europe, while other major groups are found in Central and Western European countries. Other Romani live outside of the European continent, in particular in the Middle East and in the Americas [2,8]. The Roma are a complex, structured population and considering just the national origin of its members usually fails to properly characterize this diversity. Understanding Romani structure requires the concerted efforts of many disciplines, as well as the involvement of the Romani public. For instance, the main linguistic units within the Roma are based on seven main dialect groups: Balkan, Vlax, Central, North-Eastern, North-Western, British and Iberian [11,12].

Linguistic and historical research has provided evidence that the Roma originated in the northern regions of South Asia, in particular the Punjab and Kashmiri regions of northern India [5,11]. Anthropological records have shown resemblances between the cultures of different Indian groups and Romani people [13], who have a similar social structure to the jatis of India where groups are usually defined by profession and the endogamous group constitutes the primary unit [2,14]. The origins of the Roma have been a subject of debate for a long time and hypotheses on their Indian origins were already made in the 19th century [15,16,17,18]. However, it was as a result of studies based on autosomal [5,7] and uniparental markers [19,20,21] that their genetic South Asian origin was corroborated, which was also supported by previous evidence of Mendelian mutations that are shared by the Roma and individuals of South Asian ancestry [22,23]. Because their history of migrations, bottlenecks and endogamy practices have led to the presence of a number of founder mutations [23] and sharing of unique Mendelian disorders [3,22], as well as founder mitochondrial DNA (mtDNA) and Y-chromosome (Y-DNA) lineages [6], the Roma are described as a group of founder populations with a relatively complex demography and history [21,24].

Historical records show that the Roma were initially welcomed with mixed feelings in most of the European kingdoms and became known as “Egyptians” and locally derived terms, such as ‘astigani’, ‘tsigane’ and ‘gitanos’, leading to misconceptions regarding the origins of the population. The first descriptions of the Roma included a variety of differently skilled workers, such as farmworkers, blacksmiths, craftsmen and mercenary soldiers, as well as musicians and entertainers [2,8,25]. Due to changes in the political climate, between the 15th and 16th century, the Roma were engulfed by a wave of intolerance, often forced to settle or to leave the countries in which they were travelling [26,27,28]. When Romani individuals did not accept these new imposed laws, they were arrested, enslaved, deported or killed [29,30]. Since then, and until the second part of the 19th century, they lived in slavery in many parts of western, central and eastern Europe [8,31]; the persecutions culminated into the genocide that occurred during the Second World War, in which it is estimated that the Nazi regime and its allies exterminated around half of the Romani population of Europe [32,33,34,35,36,37].

Romani people nowadays are full-right citizens in the European Union, but despite improvements in their living conditions, there is still an anti-Romani sentiment which has proven to be a difficult prejudice to overcome [38]. The Roma still suffer many inequalities, with high rates of unemployment and poverty, low participation in education and a poor health status [39,40], and governments are still slow in taking action against their discrimination [38,40].

## 2. Origins of the Roma from Genetic Evidence

The first genetic evidence of the Indian origins of the Roma came from studies based on blood group genetic markers [41,42,43] and founder mutations in the *γ-Sarcoglycan* gene [44,45]. The presence of several disease-causing Mendelian mutations [22], which can be explained by founder events, such as the congenital myasthenia caused by the *1267delG* mutation in the *CHRNE* gene, also supported the Indian origin of the Roma, as this form of inherited disorder was otherwise described only in patients of Indian and Pakistani genetic ancestry [23,46]. Other mutations, such as the *W24X* mutation present in the *GJB2* (connexin 26) gene related to non-syndromic hearing loss or the mutations present on the *LTBP2* gene, were found in the Roma [47,48,49] and are known to be present at a high frequency in a number of Indian populations [50].

However, most of the genetic evidence regarding the South Asian origin of the Roma has been provided by the analyses of uniparental lineages (mtDNA and Y-DNA), as the presence of South Asian lineages in Romani groups pointed to an Indian origin of the proto-Roma [3]. Regarding mtDNA, M haplogroups (such as M5a1, M18 and M35b), which originated in South Asia, are present in Romani groups [21]. One of the most distinct maternal lineages found in the Roma is the M5a1b1a1 haplogroup that, as expected from a founder lineage, has been observed at a high frequency but low diversity [51], and whose diversity in the Roma has been dated around 1.5 kya [51]. Other strong evidence on the Indian origin comes from Y-DNA data due to the high frequency of the Y haplogroup H1a-M82 [19,52], which is originally from North-Western India [53]. While studies based on uniparental DNA agree on the estimating dates of the origins [51,53], some have contradicted each other about the exact location within the Indian subcontinent in which the proto-Roma originated [20,21]. A study suggesting that southern India could have also contributed to the gene pool of the proto-Roma, based on the exact matches of Y-DNA H haplotypes [20], contradicted previous reports based on mtDNA and sparked an intense debate. However, as some authors noted [4], the Indian sampling of the study in question did not include groups from the north-western regions, biasing the results. More recent findings have proven that Dravidian-speaking populations from South India are also involved in the South Asian source of the Romani individuals [7], appearing to solve the contradiction regarding the identification of uniparental Roma lineages with a North-Western Indian origin and the high level of Y-STR haplotype sharing among the Roma and South Indian populations.

Based on genome-wide SNP arrays and whole-genome sequences, it has been determined that the Romani people carry approximately 20–35% South Asian ancestry [4,7], and North-West India constitutes the major source of this component [4,7,54]. The South Asian genetic ancestry is complex and can be decoupled into two components: Ancestral North Indian (ANI) and Ancestral South Indian (ASI) [55]. The former is related to the admixture events that occurred in South Asia around 2–4 kya from West Eurasian migrations [56] and, as a result, the Roma may have already carried an Ancestral West Eurasian (AWE) component derived from the South Asian ANI prior to their arrival into Europe [4,7]. The general consensus on the origins was eventually reached with the results coming from analyses of uniparental markers and whole-genome sequences, which pinpointed the origins in north/northwestern India 1.5 kya [5,6,57], in agreement with the dates obtained from uniparental markers [21,52]. Additionally, these results proved that all the analyzed Romani people descended from a common single founder population [5,6,7], as previously suggested by Kalaydjieva et al. [24].

## 3. The Romani Diaspora out of India

Historical and genetic sources agree on the fact that after the proto-Roma left North-Western India, they migrated through the Middle East, mostly through Persia (in the Iranian Plateau) and the Caucasus, Armenia in particular [4,8,13,21]. Such evidence suggests the occurrence of a southern Black Sea migration through Anatolia into the Balkans [11,58], which might account for the near absence of Arabic and the existence of a few lexical items of Georgian derivation in the Romani language [11]. At the same time, a more northerly route through the Caucasus and Crimea into Eastern Europe has been deemed as unlikely by historical sources, due to the fact that the first evidence of the Roma in Russia appears rather late, already into the 16th century [13]. Nonetheless, there is no agreement on the amount of gene flow that the Roma received during their diaspora until reaching Europe. Some genome-wide studies have suggested a long stay of Romani groups in the Middle East with substantial gene flow from local populations [59], whereas others have proposed a moderate Middle East and Caucasus gene flow during the migration across these territories [5,7]. The migration through the Middle East and Caucasus is supported by the presence of uniparental haplogroups, which most likely have a Middle Eastern and Caucasus origin, having their highest frequency in these present-day regions, such as the mtDNA U3 haplogroup in Iran and Lebanon [60] or the Y-DNA J2 haplogroup in Ingushetia and Chechnya [61]. Despite this, Middle Eastern admixture is probably overestimated due to its genetic similarity with European-related ancestry, and the presence of high-frequency uniparental haplogroups from this region in the Roma could be explained by the occurrence of high levels of genetic drift.

**Figure 1:**
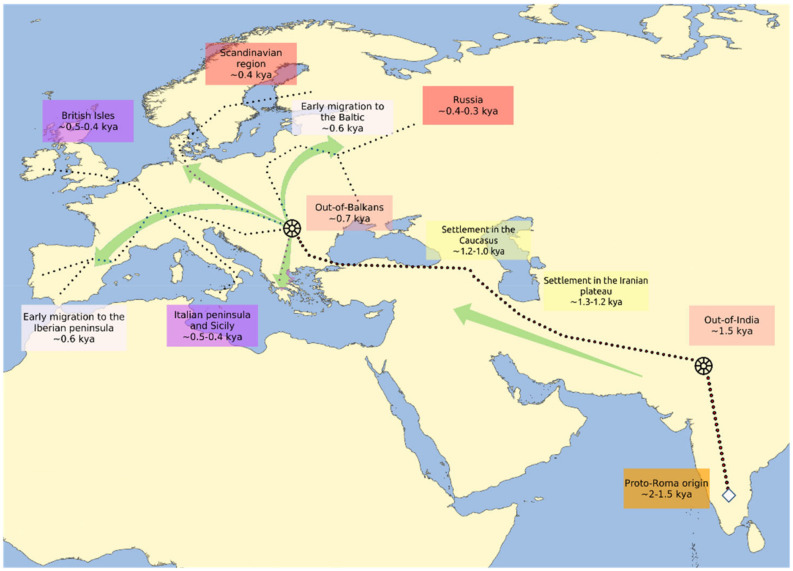
Early routes of the Romani diaspora. The figure is based on data from Kenrick 2007, Mendizabal et al., 2012, Moorjani et al., 2013, Font-Porterias et al., 2019 and Bianco et al., 2020, and reports the estimated date of arrival and settlement of the Romani populations in different regions of West Eurasia. The wheel symbols indicate the two main reported bottlenecks experienced by the Roma, the arrows indicate the main flow of the migrations and the dotted lines indicate the possible routes of the diaspora.

It has been historically suggested that the Roma reached the Anatolian peninsula between the 11th and 12th centuries [2,8,27] and the Balkans as early as the 13th century [8,31]; the dating of these events is also supported by genetic studies [4,5,7,57] (Figure 1). The Balkan genetic footprint in the Roma is well documented [4,57,59] and there is evidence of an ancestry gradient that correlates with the distance to the Balkans. In fact, the Balkan genetic component varies from 45% in Bulgarian, Greek and Serbian Roma to 25% in Baltic and Iberian Roma, which is further evidence that the dispersion into Europe took place via the Balkans [5,7]. After subsequent migrations and dispersal across Europe, Romani groups eventually reached Northern Europe and the Iberian Peninsula, the western-most part of the continent, in the 15th century [5,8]. We can speculate that one of the earlier migration waves of the Roma that started from the Balkans is the one that generated the Polish, Lithuanian and Iberian Roma [21], because only these three subpopulations show a high frequency of mtDNA U3 and Y-DNA J2 haplogroups, present in over a third of the individuals [3,51,62,63].

In general, Romani people carry approximately 65–80% West Eurasian (European, Middle Eastern and Caucasian) ancestry, estimated to have been acquired by extensive gene flow between the 13th and 16th centuries [4,7]. Among the West Eurasian sources, Eastern Europe constitutes the major source of European ancestry [4,6,7,54]. The different estimates of West Eurasian ancestry depend on the inclusion, or exclusion, of the South Asian AWE component in the Roma (derived from the ANI in Indian populations): estimates of the recent West Eurasian component increase from 65% [7] to 80% [4] when AWE is included. In addition, the influence of West Eurasian gene flow in the Roma is attested by the presence in the moderate to high frequencies of West Eurasian mtDNA (i.e., H, U, X, J, T) and Y-DNA lineages (i.e., J and R) [3,19,24,52,64].

## 4. The Genetic Differentiation and Heterogeneity among Different European Romani Subpopulations

Despite their common origin, Romani groups have experienced several population splits, founder effects, small population sizes and differential gene flow from non-Romani groups that have caused strong drift effects and rapid genetic differentiation and heterogeneity among different Romani subpopulations. The effect of this genetic heterogeneity is shown in the diverse frequency patterns of uniparental haplogroups [65,66,67,68], as well as in the frequency of disease-causing mutations [22,23,24] and genome-wide structures [4,54,57,69,70]. This has resulted in a marked genetic substructure with different levels of complexity [57,71].

**Figure 2:**
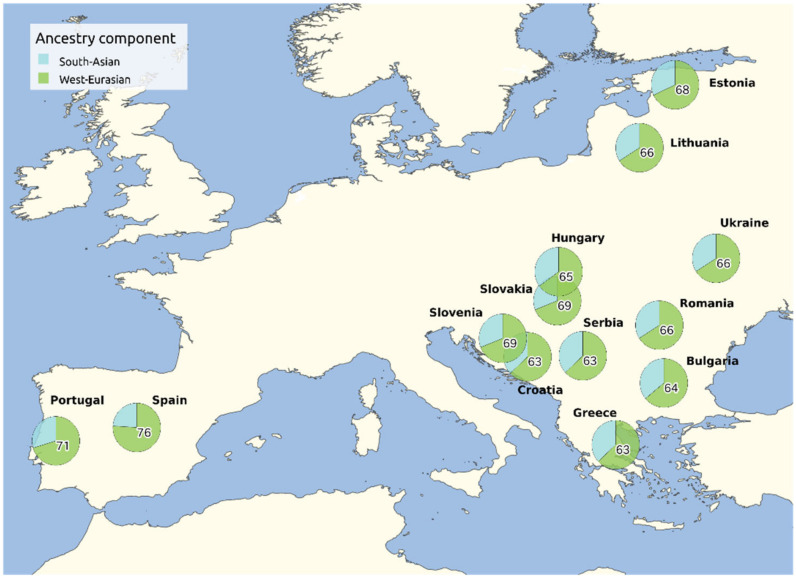
South Asian and West Eurasian ancestry components in Romani groups based on the results from Font-Porterias et al., 2019. The weighted means for the individuals obtained in the original analysis by genetic clusters are regrouped by country of origin. For sake of clarity, only West Eurasian ancestry percentages are shown.

In addition to differential bottlenecks and the loss of genetic variation experienced by each Romani group, part of the heterogeneity might be explained by different exogamy patterns expressed as gene flow within non-Romani groups, which might have been different in sources and amount [7]. As part of the recent European admixture, every Romani exhibits a Balkan common element (with different percentages in each group) and some local gene flow that are specific to each group, i.e., non-Roma Spanish into the Spanish Roma or non-Roma Polish into the Polish Roma [4,7]. The Western European Roma generally differ by exhibiting higher amounts of non-Roma European ancestry and lower levels of South Asian ancestry [4,7,72] (Figure 2).

Some differences between Romani groups can be spotted from the heterogeneous distribution of the mtDNA haplogroups and from the low frequency of M5 in the Polish, Iberian and Lithuanian Roma [3] to the presence of some exclusive branches, such as X2e, in the same Roma populations [73]. As other instances of differentiation, the Polish Roma frequently show haplotypes from W and K haplogroups that appear to be absent in other Romani populations [65], while the Romungro from Ukraine have the highest frequency of the J1 haplogroup and show a complete absence of the M5 haplogroup [6].

Differences in the time of admixture between the Roma and non-Romani groups have also been explored through the use of haplotype-based methods of dating, as these results show that the older dates of admixture for the Balkan Roma genetic clusters (a grouping method based on haplotype affinity) are around 800 ya, whereas the Iberian Roma genetic clusters are estimated to be around 600 ya [7]. The presence of South-Western European elements in the Iberian Roma also supports this hypothesis, as these elements are lacking in the Balkan groups. Nonetheless, Balkan genetic clusters, such as the Croatian Roma, show a higher level of more recent admixture, with an estimated date of admixture around 400 ya [7].

Local ancestry analyses have been used to infer more details about the time of these admixture events by measuring the length of ancestral chromosomal segments. As suggested by Mendizabal et al. [5], recent gene flow coming from non-Romani groups is supposed to generate individuals with long chromosomal segments of non-Roma European ancestry and individuals without traces of these segments, whereas cumulative recombination tends to shorten and spread the non-Roma European chromosomal tracts across the Roma population [74]. This is the case for several Romani groups from Central Europe and the Balkans, where a few individuals have shown very long ancestral chromosomal segments of non-Roma European origin; meanwhile, the populations overall have low mean values of admixture, which is possibly reflective of modern changes in social rules and endogamy practices, in particular [5]. The Roma from Lithuania and the Iberian Peninsula instead show higher values of non-Roma European admixture but in shorter segments, which might be proof of a more recent genetic isolation [5].

## 5. The Genetic Diversity of the Roma: Current Insights in the Bottleneck and Founder Events

The analysis of the demographic history of the Romani people presents two main levels of complexity. The first level of complexity comes from the gene flow with non-Romani groups that occurred all over their history, as shown in the previous sections. The second level is represented by the series of bottlenecks and splits experienced during their diaspora [5,57,75] and the different amounts of endogamy that have left traces of low intragroup diversity and high intergroup heterogeneity [6,52,63,64,65,76,77,78]. In this sense, two major bottlenecks have impacted the history of the Roma: the out-of-India and the out-of-Balkans migrations (Figure 2), events that are discernible through variations in the effective population size (*N*e), which fluctuated moderately for generations and drastically reduced in these particular instances [5,57].

According to genetic evidence, initially, the *N*e of the Roma overlapped with the *N*e of Northern Indian individuals until ~125 generations ago (ga), in agreement with the Roma origins in this region of the subcontinent [5]. The proto-Roma started to differentiate soon after the out-of-India event, proven by the occurrence of a bottleneck in which the proto-Roma *N*e became half of the parental Indian population [5,57]. It is thought that the proto-Roma generated in this circumstance left their place of origin in a single migration event [6,7]. In the period of time that goes from 125 ga to 50 ga, the Roma *N*e became lower than that of Northern Indians, and around 50 ga, it became even lower than the Dravidic groups of southern India [79,80].

The out-of-Balkans bottleneck is estimated to have happened around 900 ya, which coincides with the divergence between the Eastern and Western European Roma; the Western European Roma appear to have undergone an additional bottleneck, which reduced their *N*e to a third of that of the Eastern European Roma [5]. In the following period, both the Western and Eastern European Roma admixed with non-Roma European populations, and in a few generations, extensive gene flow from non-Roma Europeans increased the Roma *N*e, thus, compensating for the previous loss of genetic diversity [5,57]. Historically, the cessation of the *N*e reduction coincides with the settlement of the Roma in Europe and the beginning of more intense assimilation policies during the 17th century [2,57,81]. The increasing genetic distance from the Balkans and the decaying *N*e in the Western Roma suggest that cumulative drift events within Europe are one of the main catalysts of genetic differentiation within the subpopulations [5,82,83]. For this reason, Roma diversity decreased from Eastern towards Western and Northern groups due to the accumulation of drift effects during successive population splitting and migrations along the dispersion within Europe [5]. The signature of the founder effects and subsequent bottlenecks, amplified by higher levels of isolation and rates of endogamy, is also evident in the presence of a high number and total length of runs of homozygosity (RoH), in comparison to other European and Northern Indian populations [57]. In addition, a lower number of RoH was found in the Roma from the Balkans [57], possibly explicable by the fact that this group did not experience the out-of-Balkans bottleneck.

## 6. Gene Flow Sex Bias in the Roma Population

The Roma, as many human societies [84], traditionally have patrilocal residence patterns where the sociocultural group affiliation is patrilinearly inherited [85,86].

The genetic evidence for sexual asymmetries in gene flow has been provided by the data coming from studies on maternally inherited mtDNA and paternally inherited Y-DNA, due to their lack of recombination and well-established phylogeography [87]. Besides uniparental lineages, comparing differences in ancestry proportions between the X chromosome and the autosomes also provides information about sex-specific population history as two-thirds of the X chromosomes in a population are carried by women; therefore, the influence of female ancestry is higher than that of male ancestry [88]. In the case of the Roma, sex-biased patterns have been found in their genetic variation. The South Asian ancestral component in the Romani groups has been found in significantly different proportions in male and female lineages [3,6,89]. In this sense, South Asian lineages (mainly represented by the H1a1a4b2 haplogroup) have been found at high frequencies in the Y-chromosome genetic pool (approximately 50%), whereas their presence in the maternal counterpart (represented by the M haplogroups) is less frequent (less than 25%). This points to a higher male South Asian gene pool in the Romani groups [7]. Interestingly, comparing X chromosome ancestry proportions to those in the autosomes, South Asian ancestry had a higher result in the X chromosome than in the autosome [7].

Both uniparental South Asian lineages present clear evidence of founder effects [6], although the maternal lineages show higher levels of diversity, and their origin does not seem to be restricted to a single geographic location. In contrast, the Y-DNA lineages are more homogeneous, and their origin seems to be restricted to the region of Pakistan, reflecting a patrilocal pattern in the formation of the proto-Roma population [89].

Differences between males and females can also be observed in the West Eurasian lineages incorporated by the Roma during their diaspora. More than half of the mtDNA lineages in the Roma have a West Eurasian origin, belonging to different haplogroups (i.e., H7, J1b3 and J1c1) [6,21]. Some of these lineages present evidence of founder effects [6], and their origin seems to be explained by the independent assimilation of non-Roma females all along the Romani diaspora [6,21]. In contrast, the West Asian paternal lineage composition is dominated by Middle Eastern lineages with very low diversity (i.e., J2a1b). In a similar way, most European paternal lineages belong to a single haplogroup of Balkan origin (I1a-Z140) [89].

In agreement with the uniparental markers’ data, higher Middle Eastern and Caucasian ancestry proportions were observed in the autosomes when compared to the X chromosomes, suggesting a higher influx of non-Roma males in the Middle Eastern and Caucasian regions through the Romani diaspora [7].

The differences between mtDNA and Y-DNA suggest that the inclusion of non-Romani people into Roma communities has been different according to sex: non-Roma females seem to have been adopted more easily, while the low diversity levels in male lineages suggest a sporadic inclusion of non-Roma males due to changes in the socio-historical context [2,90].

## 7. Biomedical Implications of the Complex Romani Population Demography

Due to the characteristic history of the Romani population, some genetic conditions have been reported to be more frequent than in non-Romani groups. Others have been reported to be less frequent or absent, while new founder mutations have been detected, highlighting a distinct genetic background in terms of disease occurrence [22,23,91,92]. The possible impact of bottlenecks on the health of the Roma is discernible from the reduced genetic diversity (compared to other Europeans and South Asians), the depletion of rare alleles and the increased high frequency of slightly deleterious genomic variants [69,92]. The high level of homozygosity is another factor that can generate a higher frequency of harmful recessive mutations [91,92]. These demographic factors might have increased the mutational load in the Roma; however, this increase might have been counterbalanced by the extensive gene flow coming from the admixture with other populations. As a consequence, the excess of deleterious mutations is limited, as most did not have time to fixate due to rapid admixture events occurring shortly after founder events [69].

Regarding selection, several pathways related to immunity, metabolism, histone modification, cardiovascular traits, and xenobiotic response have been described to be subject to selective pressures in the Romani groups [69,93]. However, these signals of positive selection are also present in European or South Asian populations, pointing that the signals found in the Roma might be explained by selection processes that occurred before the admixture between these two groups, and that the signal is maintained in the Roma due to drift or weaker positive selection after the admixture [69].

Aside from genetic factors, previous reports suggest that the health status of the Roma is typically characterized by higher mortality, higher morbidity and shorter life expectancy (LE) than the non-Romani populations of Europe [94,95,96], where the most common causes of mortality are cardiovascular and respiratory diseases [96]. The causes of these are still unidentified and under research; however, they could likely be caused by a combination of inherited metabolic risk factors, socio-cultural and economic factors, discrimination or cultural constraints that prevent Romani people from accessing health care which has consequences, including limited access to preventive medicine [97].

As in other human populations that have experienced founder effects and bottlenecks linked to geographical, linguistic or cultural isolation, such as Finns [98], Sardinians [99] and French Canadians [100], among others, a number of private Romani mutations associated with specific single-gene disorders (Mendelian disorders) have been reported. For instance, among these private conditions, we found hereditary motor and sensory neuropathy Lom type and Russe type, congenital-cataracts facial-dysmorphism neuropathy, congenital myasthenic syndromes, limb-girdle muscular dystrophy type 2C and galactokinase deficiency to be the most common [22,23]. On average, one out of eight Romani people is a carrier of one of the five more common Mendelian mutations [22], with carrier rates for specific mutations often exceeding 5%, and sometimes 15%, depending on the analyzed group [22,23,101]. Many of the private mutations detected in the Roma have been described as ancient mutations present in the population prior to the out-of-India diaspora and are, therefore, shared by Romani patients throughout the European continent, Pakistan and India [22].

Regarding complex diseases, multiple studies have reported a higher prevalence of type 2 diabetes (T2DM) and prediabetes (PreDM) [97,102], metabolic syndrome, cardiovascular disease, a higher occurrence of obesity, overweight and hypertension [96,103], which all may contribute to their higher mortality [104]. All the risk factors were reported to be present more regularly in the Romani population compared with the majority populations in some of the studies, including smoking, sedentary lifestyle, unhealthy dietary habits, low socio-economic status and alcohol consumption, which was also associated with a higher incidence of obesity in many of them [97]. While studies have reported that the cardiovascular risk load in the Romani population differs from the non-Romani population of the same countries, the genetic risk for cardiovascular diseases appears to be in line with other European populations [105], if not lower [106]. Obesity, hypertension and smoking are also associated with metabolic syndrome, insulin resistance, high blood pressure and elevated serum lipids that can lead to the development of metabolic disorders, such as T2DM and cardiovascular disease [96]. Considering the fact that no firm conclusions can be made with the current knowledge, a metabolic hypothesis has been proposed in an effort to identify some of the causes of obesity in the Roma. The hypothesis suggests that the Roma may have experienced inadequate maternal nutrition throughout their diaspora, which would have led to fetal undernutrition. As a result, genes that would alter glucose–insulin metabolism and help the body store more energy reserves might have been selected. After settling in Europe, they acquired better nutrition and substantially lowered their energy expenditure (due to the end of their nomadic lifestyle), which may have contributed to obesity, T2DM and metabolic syndrome, all of which negatively affect LE [93,107].

For some cardiovascular/metabolic conditions, the Roma show a higher average risk allele frequency (RAF) compared to other Europeans, but the difference in RAF has so far only been found to be statistically significant only for insulin response [92]. A similar but weaker tendency was observed when comparing the Roma to Indian groups [91,92]. Generally, it is necessary to keep in consideration that Asians are genetically more susceptible to insulin resistance and diabetes than Europeans [97], and although some of the existing studies suggest a substantial prevalence of diabetes among Romani populations, and even a higher risk of developing diabetes in Romani individuals compared to non-Roma individuals, the amount of published literature on the topic remains scarce and insufficient to draw any conclusions.

In light of these aspects, it is likely that public interventions that improve general and preventive healthcare access for the Roma would have a large impact on their health conditions and life expectancy.

## 8. Future Perspectives

Despite the efforts performed in recent times to provide a complete panorama of the genetic diversity of our species and to overcome the lack of genetic data of some human populations, ethnic minorities are still underrepresented in large genomic projects [108,109]. In this sense, one important challenge in studying the population genetics of the Roma is a lack of data, the sampling biases and the underrepresentation of some of the Romani groups analyzed so far. Most population genetic studies have only considered the countries of origin of the Roma individuals, regardless of their socio-cultural and ethnic characteristics. This bias might mask part of the genetic diversity of Romani groups. Genetic data are absent for some of them, such as the scanty data from Central European Sinti groups, not to mention the complete lack of knowledge of the Middle Eastern and American Roma gene pools. In relation to social issues, it is not known to what extent socio-cultural customs are a factor in limiting gene flow and population admixture in the Roma, and sociocultural norms may promote assortative mating [110]. So far, no studies on the assortative mating of the Roma have been undertaken, and this is another matter that needs to be addressed.

Minority communities regularly experience discriminatory practices in healthcare and receive inferior medical care [111]. Therefore, as in other minoritarian populations, in contrast to the one-fits-all model, a right-treatment-to-the-right-patient approach should be sought and broader access to personalized medicine should be built. It is particularly important to evaluate how to assess the frequencies of functional genetic variants in key drug responses and metabolism genes of the Roma, as these significantly influence drug response differences in different populations [112]. To conclude, for the interpretation of ethnic inequalities, further research is required to determine the impact of how genetic, inherited, environmental effects and other environmental factors interact. As ethnicity is not only influenced by genetic ancestry but also by socio-cultural factors, a multidisciplinary approach is needed to unravel the population history of Romani groups.

Future genetic studies including Romani groups should follow the general ethical guidelines required for genetic studies of human populations, including those where minority populations participate, ensuring appropriate informed consent is obtained and data sharing policies, among other ethnic regulations, are followed [113,114]. However, some recent articles have casted a shadow over the entire field by questioning the ethical review and consenting process in studies of the Roma [115]. This patronizing attitude assumes that only members of human minorities are unable to exercise their free will when consenting to participation in a genetic project, which can be considered a form of racism. As in any other underrepresented group, the engagement of the Roma community in future genomic approaches is of pivotal importance to maximize benefits, minimize potential harms and protect the rights and interests of the participants [116,117].
